# Perceived Social Support and Psychological Stress Among Nursing Students: Evidence from a Cross-Sectional Study

**DOI:** 10.3390/healthcare14081111

**Published:** 2026-04-21

**Authors:** Bandar S. Alharbi, Majed M. Aljabri, Endale Alemayehu Ali

**Affiliations:** 1 Community and Psychiatric Mental Health Department, College of Nursing, King Saud University, Riyadh 12375, Saudi Arabia; amajad@ksu.edu.sa; 2 Department of Public Health and Primary Care, KU Leuven, Kapucijnenvoer 33, 3000 Leuven, Belgium

**Keywords:** perceived social support, perceived stress, students, nursing, education

## Abstract

**Background**: Psychological stress is a common concern among university students, which is also pronounced among nursing students due to the academic and clinical demands of their training. Persistent stress can negatively affect students’ mental well-being, academic performance, and professional development. Social support has been identified as an important protective factor. However, evidence on the relationship between perceived social support and stress among nursing students in Middle Eastern educational contexts remains limited. **Methods**: A cross-sectional survey was conducted among nursing students. The survey included the Multidimensional Scale of Perceived Social Support (MSPSS) and the 10-item Perceived Stress Scale (PSS-10), along with sociodemographic and academic characteristics. Multivariable linear regression was used to examine the association between perceived social support and perceived stress after adjusting for age group, sex, program type, living arrangement, and employment status. Differences in stress across levels of social support were also examined using analysis of variance (ANOVA). **Results**: A total of 182 nursing students participated in the study. The mean perceived social support score was 4.95 (SD = 1.42), while the mean perceived stress score was 15.49 (SD = 2.82). We found that higher perceived social support was significantly associated with lower perceived stress (β = −1.9, 95% CI: −3.4 to −0.44), indicating that a one-point increase in the MSPSS score was associated with a 1.9-point decrease in perceived stress. Other sociodemographic factors were not significantly associated with stress. ANOVA indicated significant differences in stress across social support levels (F(2,179) = 6.91, *p* = 0.001). **Conclusions**: Perceived social support was significantly associated with lower levels of perceived stress among nursing students. These findings highlight the potential importance of strengthening supportive social environments to promote psychological well-being in nursing education.

## 1. Introduction

Psychological stress is increasingly recognized as a profound public health concern that affects individuals across different stages of life [1,2]. Stress occurs when an individual perceives that the level of demand exceeds their ability to cope with resources, and this can impact not only their mental health but also their physical health [3]. Several studies have shown that prolonged exposure to stress is associated with adverse outcomes such as anxiety, depression, impaired academic performance, and reduced quality of life [4,5,6]. Among young adults, university students represent a particularly vulnerable population due to the academic, social, and financial challenges associated with higher education [7].

Studies further highlighted that university students frequently experience increased levels of psychological stress as they navigate academic responsibilities, examinations, time management demands, and transitions to independent living [8,9]. According to Beiter et al. [10], pressure to succeed, academic performance and post-graduation plans are potential actors for anxiety and depression for university undergraduate students. Thus, persistent stress during university years may negatively influence students’ academic engagement, cognitive functioning, and overall well-being, potentially leading to burnout and mental health disorders [11].

Within the broader university student population, nursing students appear to experience particularly high levels of stress due to the unique demands of their training programs and expectation to perform high in the clinical practice [12]. Combining the coursework and clinical training requires students to develop both theoretical knowledge and practical competencies in healthcare settings, while they need to make significant efforts, which might lead to stress [13]. Clinical placements further expose students to challenging environments, including direct patient care, long clinical hours, and emotionally demanding situations, which can substantially increase psychological stress [14,15,16]. A systematic review perception of stress about future work is one of the main sources of stress for nursing students [17]. Elevated stress levels among nursing students may negatively affect their academic performance, emotional well-being, and professional development.

Given the potential negative consequences of stress, identifying factors associated with reduced impact is important. One of the most widely studied factors in psychological and health research is social support [18]. According to the stress-buffering hypothesis, social support can mitigate the negative effects of stress by enhancing coping resources and promoting psychological resilience [19]. This occurs through different forms of support, including emotional support (e.g., empathy and reassurance), instrumental support (e.g., practical help in managing academic and clinical demands), and informational support (e.g., guidance and advice). Individuals who perceive strong support networks may experience lower levels of stress because supportive relationships provide emotional reassurance, problem-solving assistance, and a sense of belonging.

Perceived social support has been consistently associated with better mental health outcomes across diverse populations. Studies have shown that individuals with higher levels of perceived social support tend to report lower levels of stress, anxiety, and depression [20,21,22]. In the context of nursing education, supportive social environments may help students cope with the demanding nature of clinical training and academic responsibilities.

Although several studies have examined stress and social support among university students, relatively fewer studies have specifically investigated this relationship among nursing students in Middle Eastern contexts. Cultural factors, family structures, and educational environments may influence how students perceive social support and cope with stress. In addition, differences in clinical training demands, academic expectations, and support systems across regions may shape both the level and impact of perceived social support. Understanding these context-specific dynamics is important, as findings from other settings may not be directly generalizable. This is particularly relevant for nursing education programs, where psychological well-being is closely linked to students’ academic success and readiness for professional practice. Identifying factors associated with lower stress levels may help universities develop targeted support strategies that enhance student resilience and promote mental health.

Therefore, our study aimed to examine the association between perceived social support and perceived stress among nursing students. By exploring how different levels of perceived social support relate to students’ stress levels, this study aimed to contribute to the growing literature on student mental health and provide evidence that may inform interventions designed to improve psychological well-being among nursing students.

## 2. Methods

### 2.1. Study Design and Setting

We conducted a cross-sectional study to examine the association between perceived social support and perceived stress among nursing students at King Saud University in Riyadh, Saudi Arabia.

### 2.2. Participants and Data Collection

The study population consisted of undergraduate and postgraduate nursing students enrolled at King Saud University during the study period. Participants were recruited using a convenience sampling approach. Data were collected using an online questionnaire that students accessed through a Quick Response (QR) code. Data collection took place between 10 and 18 February 2026. The QR code allowed participants to scan and directly access the survey link using their mobile devices. The survey link was displayed on the communication board of the University. Before accessing the questionnaire, participants were presented with an electronic information sheet describing the purpose of the study, the voluntary nature of participation, and assurances of anonymity and confidentiality. Students who agreed to participate proceeded to complete the online questionnaire. Completion and submission of the questionnaire were considered as provision of informed consent. No personal identifiers were collected to ensure anonymity of the responses. Of the total 800 nursing students, 182 provided complete responses on the key variables included in the analysis, corresponding to a response rate of 23%.

### 2.3. Measures

Perceived social support was assessed using the Multidimensional Scale of Perceived Social Support (MSPSS), developed by D. Zimet et al. [23]. The MSPSS is a widely used 12-item instrument designed to measure perceived social support from three distinct sources: family, friends, and significant others. Each subscale includes four items, with responses recorded on a Likert-type scale reflecting agreement with statements about available support. Scores were calculated by averaging item responses, with higher scores indicating greater perceived social support. The questionnaire was administered in English using the original validated version. Studies reported that MSPSS has demonstrated strong reliability and construct validity across diverse populations, including university students and healthcare trainees [24,25]. Minor wording adjustments were made to improve clarity and contextual relevance, without altering the underlying constructs.

Perceived stress was measured using the 10-item Perceived Stress Scale (PSS-10) developed by Sheldon Cohen et al. [26]. The PSS-10 evaluates the degree to which individuals perceive situations in their lives as stressful during the previous month. Each item is rated on a five-point Likert scale ranging from 0 (never) to 4 (very often).

In addition to the psychological measures, participants reported several sociodemographic and academic characteristics. These variables included age group, gender, academic year, program type (undergraduate or postgraduate), and living arrangement.

### 2.4. Statistical Analysis

First, we computed scale scores for perceived social support and perceived stress according to standard scoring procedures. Internal consistency reliability of the MSPSS and PSS-10 scales was evaluated using Cronbach’s alpha coefficient.

Descriptive statistics were calculated to summarize the characteristics of the study participants. Continuous variables were presented as means and standard deviations, whereas categorical variables were summarized using frequencies and percentages.

To assess the association between perceived social support and perceived stress while accounting for potential confounding factors, multivariable linear regression models were fitted with total perceived stress score as the dependent variable and mean MSPSS score as the main determinant. We also adjusted for sociodemographic characteristics including age group (18–20, 21–23, ≥24), sex (male, female), study program (undergraduate, postgraduate), living arrangement (with family, with friends, alone, on-campus housing), and employment status (not working, per-time, full-time). We assessed the assumptions of the multiple linear regression model using standard diagnostic plots. Linearity and homoscedasticity were evaluated by inspecting residuals versus fitted values and scale–location plots. Normality of residuals was assessed using Q–Q plots. Influential observations were examined using residuals versus leverage plots and Cook’s distance. Regression results were reported as beta coefficients (β) with corresponding 95% confidence intervals.

To further facilitate interpretation of the findings and compare mean stress levels across tertiles of perceived social support, we categorized perceived social support scores into tertiles representing low, moderate, and high levels of social support. Differences in mean stress scores across these categories were examined using analysis of variance (ANOVA). When the overall analysis of variance indicated significant differences, Tukey’s post-hoc test was conducted to identify pairwise differences in perceived stress across levels of perceived social support, with adjustment for multiple comparisons [27].

## 3. Results

Of the total 182 respondents, most participants were 21–23 years old (56%), followed by 18–20 years (29%)**,** while 15% were aged ≥24 years (Table 1). The sex distribution was relatively balanced, with 94 male students (52%) and 88 female students (48%)**.** The majority of participants were undergraduate students. Regarding living arrangements, most students lived with their families. This pattern suggests that family-based living arrangements remain common among nursing students in this context. In terms of employment status, most participants were not working. The mean perceived social support score measured using the Multidimensional Scale of Perceived Social Support was 4.95 (SD = 1.42). The mean perceived stress score measured using the Perceived Stress Scale was 15.49 (SD = 2.82).

Table S1 presents the mean perceived stress scores across different levels of perceived social support. Nursing students with low levels of social support reported the highest mean stress score (16.48 ± 2.56). Students with moderate levels of social support had a lower mean stress score (15.34 ± 2.38), while those with high levels of social support reported the lowest mean stress score (14.65 ± 3.19). There is an inverse association between social support and stress among nursing students (Figure S1).

Figure S2 illustrates the distribution of perceived stress scores across different levels of perceived social support. Students with low perceived social support exhibited the highest median stress levels, with the interquartile range concentrated around higher stress scores. In contrast, students with moderate levels of social support showed slightly lower median stress levels and a broader distribution of scores. Students reporting high levels of social support demonstrated the lowest median stress scores.

Our findings showed that both instruments demonstrated good internal consistency (Table 2). The MSPSS showed excellent reliability (Cronbach’s α = 0.96), while the PSS-10 also demonstrated good reliability (Cronbach’s α = 0.89), indicating that the scales were suitable for assessing perceived social support and perceived stress in this study population.

The results of multivariable linear regression analysis on the association between perceived social support and perceived stress, adjusted for sociodemographic and academic characteristics, are presented in Table 3. Our findings showed that perceived social support was significantly associated with lower perceived stress among nursing students. Specifically, a one-unit increase in the MSPSS total score was associated with a 1.9-point decrease in perceived stress (β = −1.9, 95% CI: −3.4 to −0.44, *p* = 0.011). This reduction reflects a meaningful change in stress levels at the individual level. It suggests that even modest increases in perceived social support are associated with noticeable reductions in psychological stress among students.

We found that age group was not significantly associated with perceived stress. Compared with students aged 18–20 years, those aged 21–23 years showed a negligible difference in stress levels (β = 0.04, 95% CI: −0.94 to 1.0), while students aged ≥24 years also demonstrated no meaningful difference (β = 0.02, 95% CI: −1.6 to 1.7). These results indicate that perceived stress levels were generally similar across age groups. There was no significant difference between males and females regarding perceived stress. Male students reported slightly lower stress levels than female students (β = −0.45, 95% CI: −1.4 to 0.48), albeit they were not statistically significant. Similarly, program level showed no association with perceived stress, as postgraduate students did not differ significantly from undergraduate students in reported stress levels (β = 0.01, 95% CI: −1.8 to 1.8).

There was no significant association between living arrangement and perceived stress. Compared with students living with their families, those living with friends (β = −0.44, 95% CI: −2.7 to 1.8), living alone (β = −0.62, 95% CI: −2.5 to 1.2), or residing in on-campus housing (β = −0.22, 95% CI: −1.6 to 1.1) showed no statistically significant differences in stress levels. Employment status was also not significantly associated with perceived stress. Students working part-time tended to report lower stress compared with those not working (β = −1.3, 95% CI: −2.8 to 0.23), although the association did not reach statistical significance (*p* = 0.10). Full-time employment also showed lower stress levels (β = −0.16, 95% CI: −2.3 to 2.0), albeit they were not statistically significant.

Diagnosis plots showed that the assumptions for the linear regression model had been met (Supplementary Figure S3). The residuals against the fitted values graph revealed that the data was fairly linear, except that a slight curve could be detected. The Q-Q Plot confirmed the approximate normality of the residuals. The Scale-Location graph showed that there had been relatively equal variances throughout all fitted values.

Analysis of variance showed a statistically significant difference in perceived stress across levels of perceived social support among nursing students (F(2,179) = 6.91, *p* = 0.001) (Table 4, Panel A). This result indicates that mean stress levels differed significantly among students with low, moderate, and high levels of social support. Post-hoc pairwise comparisons using Tukey’s test further clarified these differences (Table 4, Panel B). Students reporting high levels of social support had significantly lower perceived stress compared with those reporting low levels of support (mean difference = −0.23, 95% CI: −0.37 to −0.08, *p* = 0.001). The difference between moderate and low support groups was marginally significant (mean difference = −0.14, 95% CI: −0.29 to 0.00, *p* = 0.060). However, no statistically significant difference was observed between the high and moderate support groups (mean difference = −0.09, 95% CI: −0.23 to 0.06, *p* = 0.343). These findings suggest that nursing students with higher levels of perceived social support tend to report lower levels of perceived stress, particularly when compared with students experiencing low levels of social support.

## 4. Discussion

### 4.1. Main Findings of the Study

In this study, we quantified the association between perceived social support and perceived stress among nursing students. The findings showed that students reported moderate levels of perceived stress and relatively high levels of perceived social support. Importantly, higher perceived social support was significantly associated with lower perceived stress in the adjusted regression model. In addition, students with high levels of social support reported significantly lower stress than those with low social support in the ANOVA and post-hoc comparisons. These findings indicate that perceived social support plays an important protective role in reducing psychological stress among nursing students. However, this finding should be interpreted in the context of a single-university sample, as institutional, cultural, and social factors may shape both stress experiences and perceived support. Other sociodemographic factors are not significantly associated with perceived stress. An estimate of the coefficient (β = −1.9) shows that a single-unit increase in the social support measure is related to a decrease in the PSS-10 by 1.9 points. However, the magnitude of this association should be interpreted cautiously, as the practical importance of a 1.9-point difference on the PSS-10 scale is not well established and may vary by population. The cross-sectional design further limits interpretation to association rather than effect. The ANOVA results showed consistent differences in stress levels across categories of social support, which supports the presence of an association.

In line with several previous studies, our findings demonstrate the importance of social support for people in stressful situations, such as students and healthcare trainees [28,29]. The stress-buffering hypothesis provides a theoretical explanation for this relationship [19]. According to this model, social support helps individuals cope with stressful experiences through emotional reassurance, practical assistance, and coping resources, which may reduce the negative psychological impact of stressors. However, in the current study, these pathways were not directly measured. Therefore, these explanations should be interpreted as theoretical mechanisms rather than empirically tested mediation effects. A survey-based analysis based on among 1097 college students found that social support is negatively associated with anxiety, and the association is moderated by physical exercise. In their finding, family support was the most influential factor [22]. Another study by Reeve et al. investigated perceived stress and social support among undergraduate nursing students using survey data collected through structured questionnaires. Their results showed that nursing students often experience high levels of stress, but those who reported stronger social support from peers and faculty demonstrated better coping and psychological well-being [30]. The mechanism of association is that perceived social support can affect stress via emotional support, which alleviates perceived burdens, and instrumental support, which facilitates managing academic and clinical requirements. Moreover, peer and familial social support can increase a sense of belongingness and control, both of which play a role in the process of appraising stress. These are all possible means by which social support could affect stress but are not evaluated in the current study.

Studies measure the association of perceived stress and social support in different angles. For example, a recent cross-sectional study among nursing students in Jordan used standardized instruments including MSPSS and PSS to examine the association between social support, resilience, and stress [31]. The findings indicate that there is negative association between perceived stress and social support. Another study also highlighted the negative association of stress and social support, with social support further mediating the association of stress with resilience, mindfulness and psychological well-being [32].

The negative association between social support and stress observed in our study can be justified through both psychological and social mechanisms. Social support can provide emotional reassurance and practical guidance during stressful situations, helping students interpret challenges as manageable rather than overwhelming. In academic environments, support from family, friends, and peers may also enhance students’ sense of belonging and reduce feelings of isolation, which is specifically important for nursing students as they may be overwhelmed with both course work and clinical practice. Furthermore, the finding that demographic variables such as age, gender, program level, and living arrangement were not significantly associated with stress suggests that perceived social support may be a more influential factor in determining stress levels than individual sociodemographic characteristics. This observation highlights the importance of supportive social environments within nursing education programs, without specific group of sociodemographic characteristics.

The findings of this study have several practical implications for nursing education. Universities and nursing schools should implement structured interventions to strengthen social support among students. These may include formal mentorship programs that pair junior students with senior peers or faculty, peer-support groups integrated into the curriculum, and accessible counseling services with clear referral pathways. Institutions should also consider embedding collaborative learning activities and group-based clinical debriefing sessions to enhance peer interaction and emotional support. In addition, training faculty to recognize signs of student stress and provide timely academic and emotional support may improve student well-being. These targeted strategies can help create a supportive learning environment and reduce stress among nursing students.

### 4.2. Strengths and Limitations

Our study has several strengths. We employed validated and widely used instruments, including MSPSS and PSS-10, both of which have demonstrated strong reliability and validity across diverse populations. The study used multivariable regression analysis, which allowed adjustment for several potential confounding variables such as age, sex, academic program, living arrangement, and employment status. This analysis approach strengthens the validity of the findings by isolating the independent association between perceived social support and perceived stress. Additionally, we also performed multiple complementary statistical approaches, including ANOVA, and post-hoc comparisons, providing consistent evidence for the observed relationship between social support and stress. Finally, the study contributes important evidence from a Middle Eastern academic setting, where relatively fewer studies have explored psychosocial determinants of stress among nursing students.

Despite these strengths, several limitations should be acknowledged. The cross-sectional design limits the ability to establish causal relationships between perceived social support and perceived stress. Although the results suggest that higher social support is associated with lower stress, it is not possible to determine whether social support reduces stress or whether students experiencing lower stress perceive greater support. Longitudinal studies would be required to better understand the directionality of this relationship. The use of a convenience sampling approach through QR code-based recruitment may introduce self-selection, social desirability and non-response bias, as participation depended on students’ willingness and access to the communication platform. This may limit the representativeness of the sample and reduce the generalizability of the findings. Additionally, no formal sample size or power calculation was performed, which may affect the precision of the estimated associations. Another limitation is that the study was conducted within a single university, which may limit the generalizability of the findings to nursing students in other institutions or regions. Educational environments, cultural contexts, and social structures may influence how students experience stress and perceive social support. Our study does not examine mediating mechanisms, such as resilience or coping strategies, and potential moderating effects, such as gender or living arrangement. Moderation was not tested due to insufficient sample size for reliable interaction analysis. Future studies with larger and more balanced samples should examine interaction effects to assess potential moderation. In our study, analysis of MSPSS subscales was not performed, as this would reduce statistical power and yield less stable estimates given the sample size. Finally, although several demographic variables were included in the analysis, other potentially relevant factors, such as coping strategies, resilience, and academic workload, were not examined in this study and may also influence students’ stress levels.

## 5. Conclusions

We found that higher perceived social support was significantly associated with lower perceived stress among nursing students. Students who reported greater support from family, friends, and significant others tended to experience lower levels of psychological stress. These findings highlight the importance of fostering supportive social environments within nursing education. Strengthening social support systems may help improve students’ psychological well-being and support their academic and professional development.

## Figures and Tables

**Table 1:** Sociodemographic and academic characteristics of nursing students and summary statistics of perceived social support (MSPSS) and perceived stress (PSS-10) among participants.

Characteristic	n = 182
Age group	
18–20	53 (29%)
21–23	102 (56%)
≥24	27 (15%)
Sex	
Female	88 (48%)
Male	94 (52%)
Program	
Undergraduate	161 (88%)
Postgraduate	21 (12%)
Living arrangement	
With family	143 (79%)
With friends	7 (3.8%)
Alone	11 (6.0%)
On-campus housing	21 (12%)
Employment status	
Not working	145 (80%)
Part-time	22 (12%)
Full-time	15 (8.2%)
MSPSS total	4.95 (1.42)
PSS total	15.49 (2.82)

Note: n (%); Mean (SD).

**Table 2:** Internal consistency reliability of the Multidimensional Scale of Perceived Social Support (MSPSS) and the Perceived Stress Scale (PSS-10) among nursing students (n = 182).

Scale	Number of Items	Cronbach’s α
Multidimensional Scale of Perceived Social Support (MSPSS)	12	0.96
Perceived Stress Scale (PSS-10)	10	0.89

**Table 3:** Multivariable linear regression analysis examining the association between perceived social support and perceived stress among nursing students.

Characteristic	β	95% CI	*p*-Value
MSPSS total	−1.9	−3.4, −0.44	0.011
Age group			
18–20	-	-	
21–23	0.04	−0.94, 1.0	0.09
≥24	0.02	−1.6, 1.7	0.52
Sex			
Female	-	-	
Male	−0.45	−1.4, 0.48	0.3
Program			
Undergraduate	-	-	
Postgraduate	0.01	−1.8, 1.8	0.85
Living arrangement			
With family	-	-	
With friends	−0.44	−2.7, 1.8	0.71
Alone	−0.62	−2.5, 1.2	0.52
On-campus housing	−0.22	−1.6, 1.1	0.74
Employment status			
Not working	-	-	
Part-time	−1.3	−2.8, 0.23	0.10
Full-time	−0.16	−2.3, 2.0	0.91

Note: β represents regression coefficients from the adjusted linear regression model with perceived stress (PSS-10 score) as the dependent variable. MSPSS = Multidimensional Scale of Perceived Social Support; CI = Confidence Interval.

**Table 4:** Differences in perceived stress across levels of perceived social support among nursing students.

**Panel A. Overall ANOVA Results**					
**Source**	**df**	**Sum of Squares**	**Mean Square**	**F-Value**	***p*** **-Value**
Social support level	2	102.9	51.43	6.91	0.001
Residuals	179	1332.6	7.44	—	—
**Panel B. Tukey’s Post-hoc Pairwise Comparisons Based on Standardized PSS-10**					
**Comparison**		**Mean Difference (β)**	**95% CI**	***p*** **-Value**	
Moderate support–Low support		−0.14	−0.29, 0.00	0.060	
High support–Low support		−0.23	−0.37, −0.08	0.001	
High support–Moderate support		−0.09	−0.23, 0.06	0.343	

Note: Social support level was categorized into tertiles (low, moderate, and high) based on the total score of the Multidimensional Scale of Perceived Social Support (MSPSS). Perceived stress was measured using the Perceived Stress Scale (PSS-10). Negative mean differences indicate lower stress in the group listed first. *p*-values for pairwise comparisons were adjusted using Tukey’s method.

## Data Availability

Data are not shared due to privacy and ethical restrictions.

## Supplementary Materials

The following supporting information can be downloaded at: https://www.mdpi.com/article/10.3390/healthcare14081111/s1, Table S1: Mean perceived stress scores across levels of perceived social support among nursing students. Figure S1: Association between social support and stress among nursing students. Figure S2: Perceived stress across levels of social support among nursing students. The boxplots display the median, interquartile range, and outliers of Perceived Stress Scale (PSS-10) scores for students with low, moderate, and high levels of perceived social support. Figure S3: Diagnostic plots for the multiple linear regression model.

## Abbreviations

MSPSS	Multidimensional Scale of Perceived Social Support
PSS	Perceived Stress Scale
PSS-10	10-item Perceived Stress Scale
SD	Standard Deviation
CI	Confidence Interval
ANOVA	Analysis of Variance
QR	Quick Response
GPA	Grade Point Average
