# Supplementary material for: Perceived Social Support and Psychological Stress Among Nursing Students: Evidence from a Cross-Sectional Study
Source: Healthcare (Basel). 2026 Apr 21;14(8):1111. doi: 10.3390/healthcare14081111 (PMC13115577; doi:10.3390/healthcare14081111)
Supplement: Supplementary file 1 [file healthcare-14-01111-s001.zip › healthcare-4230749-supplementary.pdf]

Supplementary Materials

Perceived Social Support and Psychological Stress Among Nursing Students: A Cross-Sectional Analysis

Bandar S. Alharbi[1*], Majed M. Aljabri[1], Endale Alemayehu Ali[2*]

[1]Community and Psychiatric Mental Health Department, College of Nursing, King Saud University, Riyadh 12375, Saudi Arabia

[2]Department of Public Health and Primary Care, KU Leuven, Kapucijnenvoer 33, 3000 Leuven, Belgium

[*] Shared corresponding author (Email: banalharbi@ksu.edu.sa; endalestat@gmail.com)

Table S1: Mean perceived stress scores across levels of perceived social support among nursing students.

Characteristic	Low support N = 61	Moderate support N = 61	High support N = 60
PSS total	16.48 (2.56)	15.34 (2.38)	14.65 (3.19)

Note: Values are presented as mean (standard deviation). Social support levels were categorized into tertiles (low, moderate, and high) based on the total score of the Multidimensional Scale of Perceived Social Support (MSPSS). Perceived stress was measured using the Perceived Stress Scale (PSS-10).

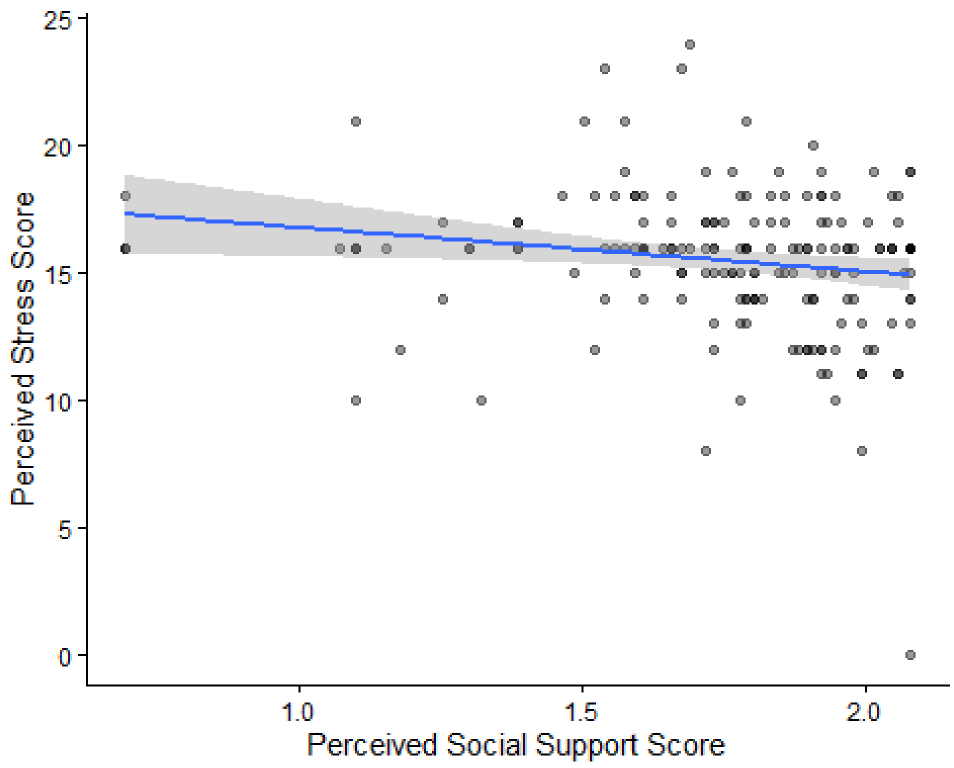

Figure S1: Association between social support and stress among nursing students

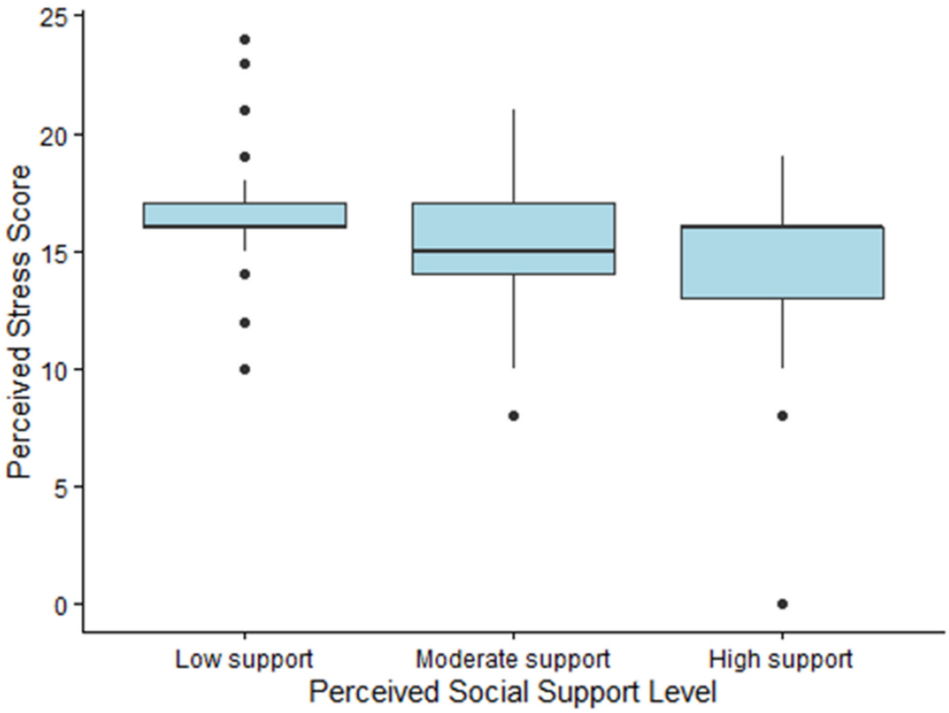

Figure S2: Perceived stress across levels of social support among nursing students. The boxplots display the median, interquartile range, and outliers of Perceived Stress Scale (PSS-10) scores for students with low, moderate, and high levels of perceived social support.

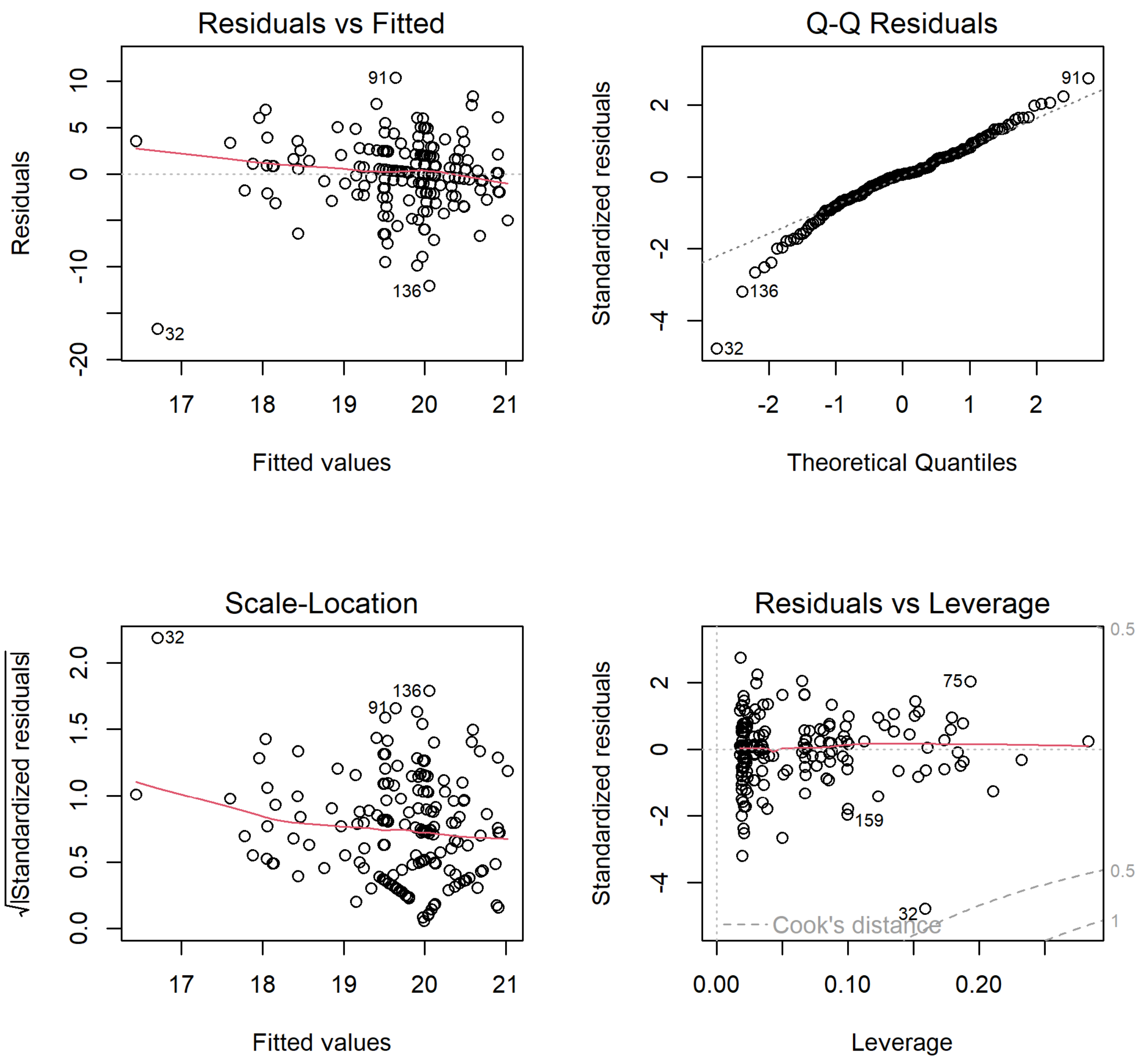

Figure S3: Diagnostic plots for the multiple linear regression model.
